# A prospective, double-blind, randomized controlled trial of treatment of atlantoaxial instability with C1 posterior arches >4 mm by comparing C1 pedicle with lateral mass screws fixation

**DOI:** 10.1186/s12891-016-1017-8

**Published:** 2016-04-14

**Authors:** Liang Yan, Baorong He, Tuanjiang Liu, Lixue Yang, Dingjun Hao

**Affiliations:** Department of Spinal Surgery, Hong Hui Hospital, Xi’an Jiaotong University College of Medicine, 555 Friendship East Road, Shaanxi, 710054 China; Department of Orthopaedics, First Affiliated Hospital, Shaanxi University of Chinese Medicine, 2 Weiyang West Road, Shaanxi, 712000 China

**Keywords:** Atlantoaxial instability, C1, Lateral mass, Pedicle, Complications

## Abstract

**Background:**

C1 posterior arch screw placement is one of the most effective treatments for atlantoaxial instability (AAI), which can be performed by either pedicle or lateral mass screw fixation. This study attempted to compare the feasibility and clinical outcomes of C1 pedicle with lateral mass screw fixations for treatment of AAI with C1 posterior arches >4 mm.

**Methods:**

A total of 140 patients with AAI (C1 posterior arches measuring >4 mm) was enrolled in this single-center, randomized, double-blind trial. The subjects were randomly assigned into two treatments: C1 pedicle (group A) or lateral mass (group B) screw fixation. The patients, independent evaluating physicians and radiologists were blinded throughout the entire study. Patients were assessed before operation and in a series of follow-ups at 6 weeks, 6 months, 1 year, and 3 years post-surgery. The operation time, volume of blood loss, intraoperative complications, bone fusion rates, Japanese Orthopaedic Association (JOA) and visual analog scale (VAS) scores were monitored.

**Results:**

All 140 patients showed overall improvements in clinical symptoms after surgery. The mean follow-up time was 24.5 ± 13.0 months. In both groups, the mean JOA scores improved significantly at the time of final follow-up as compared to prior surgery (group A: 7.1 ± 1.4 vs 13.7 ± 1.9; group B:7.3 ± 1.8 vs 13.1 ± 1.4; improvement rates: 87.2 % (group A) and 86.5 % (group B)). The VAS scores also decreased significantly in both groups at the time of final follow-up as compared to prior surgery (group A: 6.0 ± 1.3 vs 1.7 ± 0.8, and group B: 5.7 ± 1.1 vs 2.1 ± 1.2). Bone fusion was achieved within 12 months postoperatively in the patients from both groups. The operation time was significantly shorter and volume of blood loss was significantly less in the patients from group A as compared to group B (*p* < 0.01). Furthermore, thirteen patients had burst bleeding from the C1-2 venous plexus and nine patients had immediate pain and numbness in the occipitocervical region due to C2 nerve roots irritation during lateral mass screw replacement, which were not observed in the patients with C1 pedicle screw insertion. No complications such as screw loosening, shifting, breakage, or AAI were observed in both groups.

**Conclusions:**

C1 pedicle screw fixation is less invasive and simpler, and has fewer complications. It renders better clinical outcomes than lateral mass screw fixation for treatment of AAI.

**Trial registration:**

Current Controlled Trials ChiCTR-IOR-15006748.

## Background

Atlantoaxial instability (AAI) is an abnormal movement at the junction between the atlas (C1) and axis (C2). AAI is resulted from pathological alterations of either bone or ligament which can be caused by congenital malformations, trauma, neoplasms, and inflammatory disease, etc. An atlantoaxial fixation is required in symptomatic AAI in order to repair the deformity, restore vertebral stability, and reduce neurological consequences [1, 2]. Various approaches have been developed and used for atlantoaxial fixation, such as sublaminar wiring, Harms and Magerl techniques. However, those methods have many disadvantages yet to be overcome. For example, sublaminar wiring renders poor primary stability and bone grafting, which requires postoperative immobilization for a long period. Patients are also burdened with a considerable rate of nonunion. Harms and Magerl are generally used for posterior C1–C2 fusion but inappropriate for patients with fixed subluxation of C1 and C2, as well as have risk of vertebral artery injury. In the Harms technique, polyaxial screws are independently inserted into the C1 posterior arch and C2 pedicles, which are connected by a small-diameter rod. Therefore, C1 posterior arch screw fixation techniques can be divided into pedicle and lateral mass screw fixation [3–6]. This study attempted to compare the feasibility and clinical outcomes of C1 pedicle with lateral mass screw fixation for treatment of AAI with C1 posterior arches >4 mm.

## Methods

### Patient population

This study was approved by the Institutional Review Boards and the Ethics Committee of the Hong Hui Hospital, Xi’an Jiaotong University. A written informed consent was obtained from every patient after a full explanation of the therapeutic procedure was instructed. A total of 140 patients with AAI were enrolled in this prospective, double-blind, single-institute, randomized controlled study between January 2007 and January 2013. The subject pool comprised 88 male (62.9 %) and 52 female (37.1 %) with a mean age of 44.5 ± 8.6 years (range, 14–59 years). All patients had various degrees of neck and occipital pain, activity limitation, numbness of limbs, or movement disorders. The inclusion criteria were AAI or reducible dislocation resulting from trauma, congenital malformation, or inflammatory. The exclusion criteria were irreducible atlantoaxial dislocation, tumor, C1 posterior arches measuring ≤ 4 mm and severe osteoporosis. An Anderson type II chronic odontoid fracture was present in 36 cases, type III in 43 cases, congenital isolated odontoid abnormalities in 34 cases, transverse atlas ligament rupture in 19 cases, and atlantoaxial dislocation caused by rheumatoid arthritis in 8 cases.

### Preoperative preparation

Cervical anteroposterior, lateral, open mouth X-rays, 3D-Computed Tomography (3D-CT) and magnetic resonance imaging (MRI) were performed for all patients. All slice CT scans were reviewed preoperatively to evaluate the atlas anatomy, screw size acceptance and placement feasibility. Skull traction was occasionally performed preoperatively with a traction weight of 3 to 5 kg. Bedsides periodical radiographies, the traction weight and angle were adjusted according to the reduction conditions. The patient combined with spinal cord injury in 8 h was treated by methylprednisolone pulse therapy.

### Surgical procedure

**Fig. 1:**
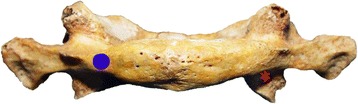
Entry points of atlas pedicle screw (*blue dot*) and atlas lateral mass screw (*red asterisk*)

Patients were assigned into two groups using a computerized random number generator: group A (C1 pedicle screw fixation) and group B (C1 lateral mass screw fixation). Each patient was placed in the prone position under general anesthesia, neck was immobilized in a neutral position and skull traction was maintained until plate placement. A midline incision was made to expose the posterior elements of C1–C3. The medial and lateral margins of the lateral mass of the axis and the posterior surface of the posterior lamina of the atlas were then dissected. The entry point for the C1 pedicle screw was 18–20 mm lateral to the posterior tubercle of the atlas and 3 mm inferior to the superior border of the posterior arch, under the vertebral artery groove. The screw direction was a mean medial inclination of 10° and rostral direction of 5°. The starting point for lateral mass screw insertion was at the intersection of the inferior border of the C1 posterior arch and the midpoint of the C1 lateral mass. The screw direction was a mean medial inclination of 10° and rostral direction of 15°. All operations were performed by two senior orthopedists specialized in spine surgery (Fig. 1).

### Follow-up and comparison parameters

Postoperative external immobilization via hard collar was used for 3 months. Patients were followed up at 3 months, 6 months postoperatively and every 6 months thereafter, with clinical examinations, X-ray and 3D-CT.

The operation time, volume of blood loss and intraoperative complications (venous plexus injury, vertebral artery injury, and spinal cord injury) were monitored for the patients in each group. At each follow-up, the JOA score, VAS score, and bone fusion rate were recorded. The patients, evaluating physicians, and radiologists were blinded throughout the entire study.

### Statistical analysis

Data of demographic, radiographic parameters and clinical outcomes of surgery were analyzed using the SPSS statistical software for Windows V13.0 (SPSS Inc., Chicago, IL, USA). The paired *t* test was used for two-group comparisons. Data are presented as the mean ± standard. For all analyses, a *p* value of < 0.05 was considered statistically significant.

## Results

**Fig. 2:**
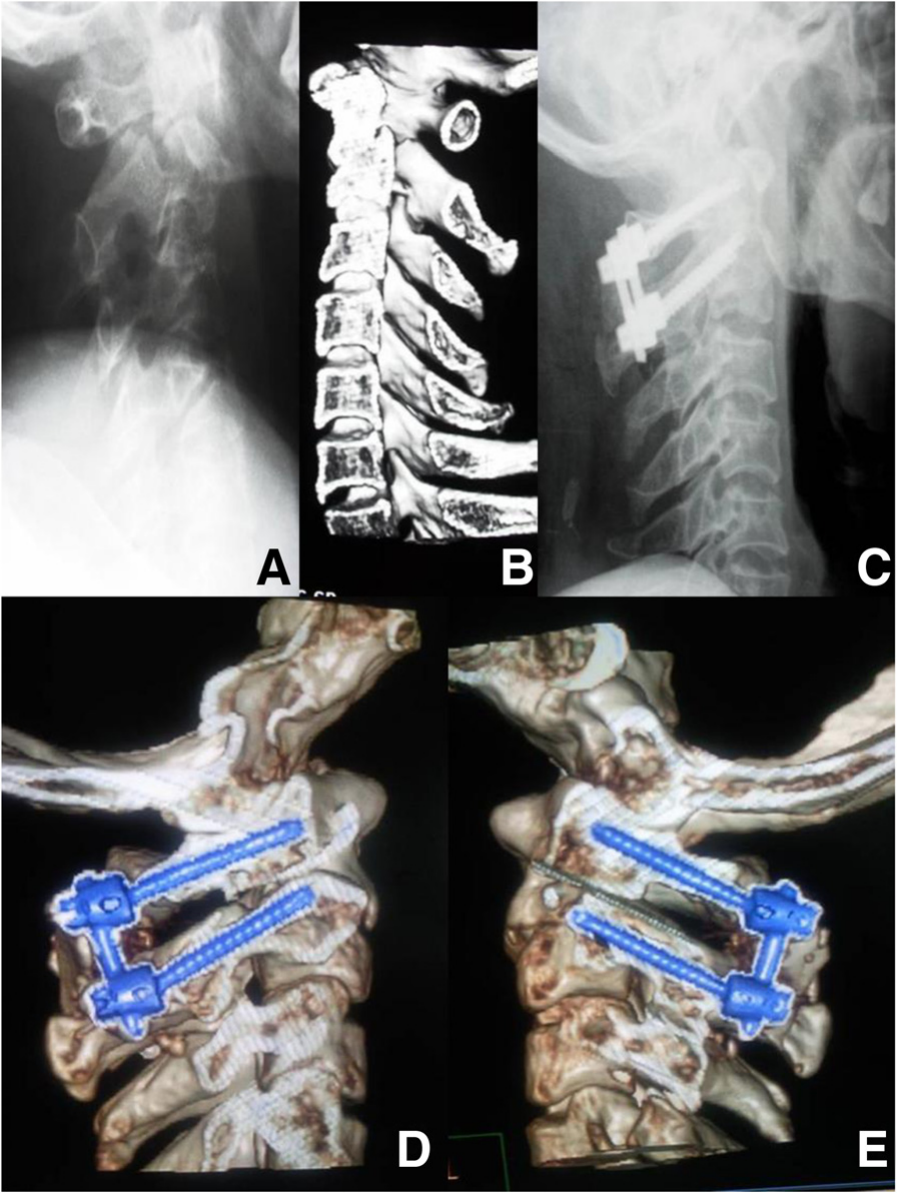
A patient who presented with an odontoid fracture underwent atlantoaxial fusion and internal fixation. **a** and **b** Lateral X-ray and sagittal reconstruction CT scans showing odontoid fracture. **c** Lateral X-ray after surgery revealing good fracture reduction and internal fixation. **d** and **e** Postoperative sagittal reconstruction CT scans showing atlantoaxial internal fixation with atlas pedicle screw and axis pedicle screw

**Fig. 3:**
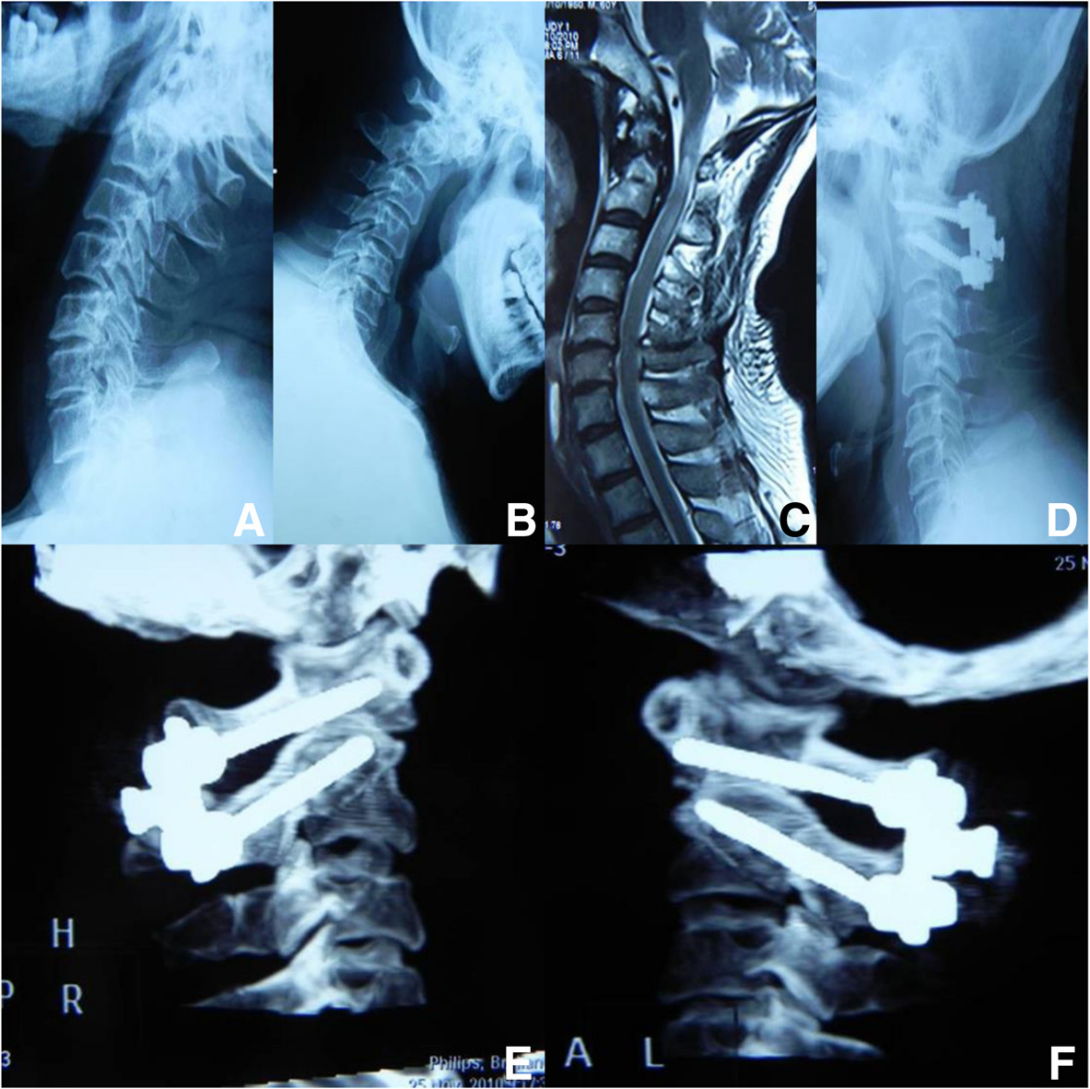
A patient who presented with atlantoaxial instability and cervical spinal cord injury underwent atlantoaxial fusion and internal fixation. **a** and **b** Dynamic radiographs showing atlantoaxial instability. **c** On MRI, the cervical spinal cord showing a hyperintense signal on T2-weighted images. **d** Lateral X-ray after surgery revealing good fracture reduction and internal fixation. **e** and **f** Postoperative sagittal reconstruction CT scans showing atlantoaxial internal fixation with atlas lateral mass screw and axis pedicle screw

Screw placement was successfully completed in all patients without radiographic assistance (Figs. 2 and 3). There were no differences in age, sex, body mass index, clinical symptoms, JOA score, ASIA grade, VAS, or follow-up period between the group A (*n* = 67 subjects) and B (*n* = 73 subjects) (Table 1). The mean operation time in group A was significantly shorter than group B (85 ± 11 vs 110 ± 17 min, *p* < 0.01). The estimated volume of blood loss was significantly less in the patients in group A than group B (180 ± 40 vs 370 ± 80 ml, *p* < 0.01) (Table 2).

**Table 1:** Characteristics of the patients

	Group A	Group B	*P* value
Number of patients (n)	67	73	
Sex (M/F)	43/24	45/28	0.812
Age (years)	43.9 ± 8.2	45.2 ± 8.7	0.366
Mean body mass index (kg/m^2^)	24.2 ± 3.0	24.5 ± 2.7	0.535
JOA score	7.1 ± 1.4	7.3 ± 1.8	0.467
VAS score	6.0 ± 1.3	5.7 ± 1.1	0.142
Mean follow-up(months)	24.8 ± 13.6	24.4 ± 11.8	0.853

*JOA*, Japanese Orthopaedic Association; *VAS*, visual analog scale

**Table 2:** Comparison of intraoperative indexes between two groups

	Operation time	Estimated blood loss	Venous plexus injury	Nerve root injury
Group A	85 ± 11	180 ± 40	0	0
Group B	110 ± 17	370 ± 80	13	9
*P* value	<0.01	<0.01	<0.01	<0.01

### Clinical results

The patients in both groups had similar lengths of hospitalized time (9.8 ± 1.2 vs 10.1 ± 1.9 days in the group A and B, respectively). All patients were followed up from 12 to 38 months (average: 24.5 ± 13.0 months) and showed some extent of postoperative improvements in clinical symptoms. The JOA scores of patients in the group A improved from 7.1 ± 1.4 prior operation to 13.7 ± 1.9 at the time of final follow-up (*p* < 0.01). The postoperative improvement rate was 87.2 % (range, 78.6 %–92.6 %). Similarly, the mean JOA scores of patients in the group B improved from 7.3 ± 1.8 prior operation to 13.1 ± 1.4 at the time of final follow-up (*p* < 0.01), with the postoperative improvement rate as 86.5 % (range, 76.7 %–93.2 %). There were significant decreases in VAS scores in the patients in both groups measured before operation as compared with at the time of final follow-up (6.0 ± 1.3 vs 1.7 ± 0.8 (group A), 5.7 ± 1.1 vs 2.1 ± 1.2 (group B), *p* < 0.01). There were no statistical significances in the scores of VAS and JOA between the two groups at any time points throughout the preoperative period to the postoperative follow-up series (Table 3).

**Table 3:** Comparison of postoperative clinical outcomes between different groups

	Group A			Group B		
	preoperative	postoperative	*P* value	preoperative	postoperative	*P* value
JOA	7.1 ± 1.4	13.7 ± 1.9	<0.01	7.3 ± 1.8*	13.1 ± 1.4*	<0.01
VAS	6.0 ± 1.3	1.7 ± 0.8	<0.01	5.7 ± 1.1*	2.1 ± 1.2*	<0.01

*JOA*, Japanese Orthopaedic Association; *VAS*, visual analog scale

*compare with same index of Group A, *P* > 0.05

### Radiological results

Overall, no hardware-related complication was observed in any patient throughout the postoperative follow-up series. CT scans at 12 months post- surgery confirmed that fusion was achieved in all patients. However, six patients with an inferior wall fracture of the posterior arch, two with penetration of axis pedicle screws into the vertebroarterial foramen, and two with a medial wall fracture of the axis pedicle, were noted.

### Complications

Thirteen patients in the group B had burst bleeding from the C1-2 venous plexus during surgery. Nine patients in the same group had immediate pain and numbness in the occipitocervical region caused by C2 nerve roots irritation. The pain disappeared in four patients and persisted in other patients. No other major surgery-related complications were observed, including wound infection, additional neurological dysfunction, or hardware failure.

## Discussion

The dens and ligamentous structures are critical to maintain the stability of atlantoaxial joints. Various pathological conditions that lead to abnormalities of these structures will result in AAI, which an atlantoaxial fixation will be required to repair vertebral deformity, restore their stability and avoid neurological consequences [2, 4]. Many surgical approaches have been developed and applied clinically to treat AAI, including posterior interspinous fusion with sublaminar wires, iliac bone grafts, interlaminar clamps, C1-C2 transarticular screw fixation and posterior atlantoaxial fixation with polyaxial screws and rods. However, each one has its own limitations and disadvantages. For instance, sublaminar wires and interlaminar clamp techniques have a risk of spinal cord or vertebral artery injury, requiring posterior vertebral elements intact, long term postoperative Halo immobilization, and rotational and translational motion. Another example is transarticular screw technique, which provides good stability in rotatory motion and has no requirement of posterior vertebral elements intact, with drawbacks of anatomical variations such as irreducible C1-C2 subluxation. The recent posterior transpedicular screw fixation techniques render favorable biomechanical properties and wide indications, which have been gaining increasingly attention by spine surgeons [2–4, 6–8].

The ring shaped first cervical vertebra, namely the atlas (C1), has different anatomical features from other cervical vertebrae in lack of a vertebral body and spinous process. It is formed by an anterior and posterior arches attached to two lateral masses [5, 7, 9–12]. The principle of posterior C1 screw fixation techniques is composed of: (1) vertebral stabilization by C1 lateral mass screw, which is inserted directly into the lateral mass of C1 via the inferior base of the posterior arch; and (2) fixation by C1 pedicle screw, which is placed through the posterior arch into the lateral mass of C1 [6, 10, 11]. A greater stability can be achieved by the C1 pedicle than lateral mass screw due to its longer trajectory [10]. As for the complexity of surgical techniques and vulnerability of enriched vertebral neuro-vascular networks, skilled surgeons with good knowledge about the anatomy of vertebrae and their vicinity, especially the anatomic interfaces between the vertebral artery and C1-C2 vertebrae, are crucial for a successful screw fixation treatment of AAI [13].

The vertebral artery, with multiple loops and an intimate relationship with the atlas and axis bones, travel within craniovertebral channels in a serpentine course. While the venous plexus that cover the entire course of the vertebral artery makes identification possible during the surgery. The vertebral artery ascends relatively linearly in the foramen transversarium of C6 to C2 where it exits and takes a loop. It then travels within the vertebral artery groove over the lateral aspect of the posterior arch of the atlas, which is vulnerable for an injury during a posterior midline approach [8, 12–16]. Our previous study has shown that the vertebral artery and vertebral artery groove are on an average distance of 17.8 and 22.3 mm away from the midline, respectively [11]. The C1 roots travel inferiorly and are posterior to the vertebral artery during their courses over the posterior arch of the atlas. In the present study, a total of 67 C1 pedicle screws and 73 C1 lateral mass screws were successfully inserted in patients in the groups A and B, respectively. Selection of specific entry points usually led to determining different exposure regions of the posterior arch of the atlas during operations. The entry point of C1 pedicle screw was positioned at 18 to 20 mm lateral to the posterior tubercle of atlas and 3 mm inferior to the posterior arch under the vertebral artery groove. The entry point for the lateral mass screw was positioned inferior to the posterior arch, even in some patients with an undersized atlas pars interarticularis. Therefore, the entry point of C1 pedicle screw had a high probability of artery injury due to its close position to the superior margin of posterior arch. In order to avoid the possible injury to the vertebral artery, careful and extensive subperiosteal dissection is required during the exposure process. However, a wide exposure of the lateral joint is necessary for insertion of lateral mass screw, which has a high risk to damage the engorged vulnerable venous plexus. In the present study, no patient had a vertebral artery injury in the group A, but thirteen patients in the group B had burst bleeding from C1-C2 venous plexus. Although the bleeding was resolved by gelatin sponge compression, the operative procedure was seriously interfered by the fuzzy field.

In order to minimize the risks of screw loosening, shifting, breakage or AAI, a tailored procedure to each individual is advised based on measurement of the atlas on preoperative 3D-CT scans [13]. In our study, the preoperative 3D-CT scans were performed in each patient to help to determine an operative approach. In postoperative radiographs and 3D-CT examinations, six patients showed an inferior wall fracture of the posterior arch. Bone fusion was achieved without screw loosening, shifting, breakage or AAI at 12 months post-surgery. Symptoms such as postoperative neck pain improved significantly for all patients, with significant decreases in the VAS scores. However, nine patients had suspected C2 nerve root irritation, probably due to excessive traction during lateral mass exposing. Improvements were observed in four patients; while others needed long-term usage of anodyne.

There are different learning curves for placing screws at C1 through pedicle or into lateral mass. So far, there is no report regarding to analyzing the learning curve of C1 screw insertion. The perforation rates of C1 pedicle and lateral mass screw in our previous study were 15.1 % and 13.0 %, respectively [11]. In this study, the perforation rate of pedicle screw dropped to 8.9 % (6 out of 67 screws) and the lateral mass screw was zero. Assessment of learning curve should be standardized by taking account of procedure time, volume of blood loss, reoperation rate and complications’ occurrence, etc. In addition, anatomical features shall also be considered. For example, the height of posterior arch at the vertebral artery groove bottom is sometimes very important for pedicle screw insertion [13, 17, 18]. Moreover, surgeons with more time related and hands on experience to insert screw will have better accuracy. Therefore, cervical spine surgeons, who have received thorough trainings and had good experiences, can make C1 screw insertion just as safe as those with assistance from a navigation system. However, the risk in early periods of learning curve cannot be underestimated.

This study has several strengths. To the best of our knowledge, this is the first double-blind, randomized controlled trial to compare the C1 pedicle screw with lateral mass screw for the treatment of AAI. The patient series was consecutive and all underwent operations performed by the same surgeons, for eliminating inter-surgeon variability and allowing for assessment of the learning curve. However, there are several limitations in this study. First, the enrollment number of this study was relatively small. As more cases are performed using this technique, its safety and efficacy can be more thoroughly evaluated. Second, this study included patients with an age range of 14 to 59 years. There were three patients under 25 years of age who were skeletally immature adolescents, which had different morphology and anatomy of the cervical spine from adults. Third, a longer follow-up would be necessary to evaluate the long-term clinical outcomes between the two groups.

## Conclusion

In conclusion, we have shown that C1 pedicle screw fixation is less invasive with simpler procedure and has fewer complications than lateral mass screw for the treatment of AAI. However, the long-term clinical outcomes are yet to be determined. To lessen the screw perforation rates during their early training stages, less-experienced surgeons must be assisted by skilled cervical spine surgeons, therefore should avoid the dangers of lethal and/or severe complications.
